# Factors Associated with COVID-19 among Healthcare Workers in Kedah in 2021: A Cross-Sectional Study

**DOI:** 10.3390/ijerph192315601

**Published:** 2022-11-24

**Authors:** Rosidah Omar, Maznieda Mahjom, Nur Haryanie Haron, Rosmanajihah Mat Lazim, Fadhlin Saffiya Qistina Kamal

**Affiliations:** 1 Occupational and Environmental Health Unit, Kedah State Health Department, Jalan Kuala Kedah, Simpang Kuala, Alor Setar 05400, Kedah, Malaysia; 2 Centre for Occupational Health Research, Institute for Public Health, Blok B5 & B6, Kompleks NIH, No. 1, Jalan Setia Murni U13/52, Seksyen U13 Bandar Setia Alam, Shah Alam 40170, Selangor, Malaysia

**Keywords:** healthcare workers, healthcare-associated, COVID-19, exposure, health systems

## Abstract

This study aimed to examine the characteristics of HCWs infected with COVID-19 and factors associated with healthcare-associated infection. A cross-sectional study, using secondary data of COVID-19 HCW cases from a registry developed by the Occupational and Environmental Health Unit (OEHU) in Kedah State Health Department, Malaysia, was analysed using Excel and STATA version 14.0. Descriptive analysis and multiple logistic regression were conducted to identify the factors for healthcare-associated COVID-19 infection. A total of 1679 HCWs tested positive for COVID-19 between 1 January 2021 and 19 September 2021. The infection was mainly non-healthcare-associated (67.0%), with healthcare-associated cases contributing to only 33% of the cases. The significant factors associated with healthcare-associated transmission were the following: doctor (aOR = 1.433; 95% CI = 1.044, 1.968), hospital setting (aOR = 1.439; 95% CI = 1.080, 1.917), asymptomatic (aOR = 1.848; 95% CI = 1.604, 2.130), incompletely or not vaccinated (aOR = 1.400; 95% CI = 1.050, 1.866) and CT-value ≥ 30 (aOR = 2.494; 95% CI = 1.927, 3.226). Identifying factors of healthcare-associated infection would help in planning control measures preventing healthcare-associated transmission in the workplace. However, more than half of COVID-19 cases among HCWs involved non-healthcare-associated COVID-19 infection, and, thus, requires further study to identify high-risk behaviours.

## 1. Introduction

There was an outbreak of Coronavirus disease (COVID-19), an infectious disease, in Wuhan, China, in December 2019, with an unknown aetiology, which, since then, has rapidly spread worldwide. This disease was caused by a novel coronavirus, Severe Acute Respiratory Syndrome Coronavirus 2 (SARS-CoV-2), an infective agent which is highly transmissible via inhaling droplets and small particles containing the virus [1]. People with COVID-19 positive may develop wide ranging symptoms from mild to severe. Common symptoms are fever, chills, cough, shortness of breath, fatigue, headache, sore throat, nausea and vomiting, and runny nose [2]. The disease caused clusters of severe respiratory illness similar to severe acute respiratory syndrome coronavirus and was associated with Intensive Care Unit admission and high mortality [3]. Due to the rapid acceleration of recorded COVID-19 cases, the disease became a threat to global health, and the World Health Organisation (WHO) declared the outbreak to be a pandemic in March 2020 [4]. As of 4 April 2022, a total of 489,779,062 confirmed COVID-19 cases, including 6,152,095 deaths, have been recorded. In Malaysia, there were 4,256,469 confirmed COVID-19 cases with 35,127 deaths reported by the Ministry of Health, Malaysia, as of 4 April 2022 [5].

The rampant spread of COVID-19 created fear and panic among people, and, hence, requiring every country to take urgent and aggressive action against the virus. In April, 2022, the WHO provided some preventive measures for the public to protect themselves and prevent the spread of the virus. These measures included practising social distance, wearing a properly fitted mask, keeping good hygiene, washing hands with soap and water frequently, covering mouth and nose when coughing or sneezing and getting vaccinated to lower the risk of getting infected [6]. The efforts to fight COVID-19 required responsibility from every individual.

According to Joseph and Joseph, “a healthcare worker is one who delivers care and services to the sick and ailing either directly as a doctor and nurse or indirectly as an aide, helper, laboratory technician, medical assistant, attendant or even medical waste handler” [7]. Healthcare workers (HCWs) were the integral frontliners in combating COVID-19. They were not only classed as high risk in terms of contracting the infection, but also in terms of their ability to spread the virus to vulnerable patients, their colleagues and their own family members. Chutiyami et al. mentioned, in their article, that the infection rate of COVID-19 among HCWs ranged from 3.9% to 11% and the highest rate involved screening activity of the HCWs [8]. In addition to its impact on morbidity and mortality, COVID-19 in a healthcare facility was also associated with increased work absenteeism and disruption of healthcare services, as well as high expenditure on treatment, contact-tracing and infection-control measures.

A study conducted in Singapore reported 1.7% of HCWs contracted COVID-19 infection and the majority of transmissions resulted from family/household exposure and social interactions [9]. Similarly, Joash and Brian reported two HCWs in Hospital Teluk Intan Perak (HTI) contracted COVID-19 infection while attending a social event, yet the infection spread among another 45 HCWs at work [10]. The contributory factors for the workplace transmission were described as enclosed and crowded places and lack of enforcement of social distancing and wearing of surgical masks at that point of time.

This study looked into the cycle threshold value, commonly referred to as the Ct-value, of the healthcare workers that were infected with COVID-19. The Ct-value represents the number of nucleic acid amplification cycles that occurred before a specimen containing the target material generated a signal greater than the predetermined positivity threshold. Generally, the higher the Ct-value, the lower the quantity of viral load present in the sample. A Ct-value of more than 30 is usually considered to be a high Ct-value and signifies low viral load. A high Ct-value may also indicate that the exposure was old and the virus isolation later, as compared to the symptom onset. Singanayagam et al. reported that, in the first week of symptom onset, the geometric mean (GM) of the Ct-value was 28.18, in the second week (days 8 to 14) the GM Ct was 30.65 and after 14 days the GM Ct was 31.60 [11]. Therefore, risk assessment and contact tracing should be conducted as early as possible for early screening and isolation to prevent healthcare transmission of COVID-19.

In Malaysia, evidence on the characteristics of COVID-19 infection among HCWs, and the factors associated with healthcare-associated infection, is understudied. Hence, this study aimed to examine the characteristics of HCWs infected with COVID-19 infection and to determine the factors associated with healthcare-associated infection.

## 2. Materials and Methods

### 2.1. Study Type, Design and Data Collection

This is a descriptive cross-sectional study using secondary data from the COVID-19 cases registry that was developed by the Occupational and Environmental Health Unit (OEHU) in Kedah State Health Department, Malaysia [12]. The information on exposure variables included socio-demographic, workplace and clinical characteristics.

The study population included all HCWs in Kedah confirmed as having COVID-19 infection during the study period of 1 January 2021, till 19 September 2021. The inclusion criteria were being a HCW, trainee or health facilities support service worker in the public and private health care facilities in Kedah. The data was collected by reviewing the HCW COVID-19 positive registry and its investigation form for each case.

Positive cases among HCWs were classified as either healthcare-associated or non-healthcare-associated and both groups were compared in terms of age, gender, ethnicity, comorbidity, workplace setting, reason for screening, symptoms, vaccination status, managing COVID-19 and level of Ct-value.

Healthcare-associated infection is defined as infection among HCWs with not wearing appropriate Personal Protective Equipment (PPE), according to the guidelines mentioned in Annex 8, Tables 5 and 7 by the Ministry of Health Malaysia, which includes infection due to providing direct care for COVID-19 patients, working with health care workers infected with COVID-19, visiting patients or staying in the same closed environment as a COVID-19 patient. Non-healthcare-associated infection is due to household contact or social contact exposure, defined as living in the same household as a COVID-19 case or working in close proximity to, or travelling in any kind of conveyance with, a COVID-19 case [13]. There is a possibility that the source of infection could be both healthcare-associated infection and community acquired. In this case, thorough investigations were conducted by the health officers and the head of department/the public health physician of the respective unit to determine which source of infection was prominent, by investigating the time and period of exposure to positive patients, symptom onset and level of CT value to minimize the overlapping of outcome.

The vaccination status of the infected HCWs was categorized in terms of the following: (1) Completed vaccination were those who received a vaccination two weeks after their second dose in a two-dose series, more than 28 days after receiving a single dose for a single-dose vaccine or receiving a booster dose for those who had Sinovac; (2) Incomplete vaccination were those who violated this definition [14]. The date of completed vaccination was compared with the date of COVID-19 diagnosis. The completed vaccination group was considered to refer to an infection that occurred after completed vaccination, whereas incomplete vaccination was an infection that preceded completed vaccination.

The reasons for screening consisted of the following: (1) Contact with family member/friend; (2) Contact with patients/work colleagues; (3) Symptomatic screening due to HCWs having fever, runny nose, cough, anosmia, ageusia, etc.; (4) Targeted screening, which was mass screening due to health facility clusters, pre-placement of new staff, pre-hospital admission, or living in an Enhanced Movement Control Order (EMCO) area.

Data were checked for missing values and duplication. All 1679 cases had complete data except 164 (9.8%) who had a missing value for the variable of Ct-value. There was no duplication, hence all 1679 cases were subjects for analysis.

### 2.2. Statistical Analysis

All the analyses were performed using statistical software, STATA version 14 (StataCorp LP, College Station, TX, USA). Descriptive statistics were used to describe the socio-demographic characteristic of the study population. Comparisons between the healthcare-associated and non-healthcare-associated cases were performed using the chi-square test or Fisher exact test to measure the association between the socio-demographic characteristics, vaccination status, workplace characteristics and clinical characteristics with the exposure status. Variables with a *p*-value of less than 0.25 in the bivariate analysis were included and were further analysed in the univariate logistic regression. This was to retain important confounding variables for a potentially slightly richer model. Variability of reasons for screening was not included in the univariate analysis, since it was part of the criteria of the outcome measured, and, hence, it violated the assumption of the logistic regression [15].

The factors for healthcare-associated COVID-19 were modelled by using multivariate logistic regression. The statistical significance was set at *p* < 0.05. To assess the goodness of fit, the Hosmer–Lemeshow test, classification table and area under the receiver operating characteristics (ROC) curve were tested [16].

## 3. Results

As tabulated in Table 1, our findings showed that among 1679 positive COVID-19 HCWs, 164 (9.8%) HCWs had missing Ct-value data. The majority of the cases were in the age group of less than 40 years of age (1154, 68.7%), female (1142, 68.0%) and Malay (1495, 89.0%). Only 264 (15.7%) HCWs with COVID-19 infection had comorbidities, commonly pregnancy in female HCWs (5.6%), hypertension (5.5%), diabetes mellitus (3.4%), asthma (2.6%) and heart disease (1.1%).

Most of the HCWs were nurses (618, 36.8%), followed by ancillary staff (406, 24.2%) and doctors (291, 17.3%). Most of the participants worked in a hospital setting (1258, 74.9%).

The reasons for screening were when the cases were epidemiologically linked to exposure from family members or friends (469, 27.9%), patients or work colleagues (489, 29.1%), symptomatic screening (616, 36.7%), and targeted screening (105, 6.3%).

The cases of COVID-19 infection were mainly non-healthcare-associated, 1125 (67.0%), whereas only 554 (33%) of the cases were healthcare-associated cases. The results presented significant association (*p*-value < 0.05) between healthcare-associated infection with age group, ethnicity, comorbidity, job category, workplace setting, reason for screening, vaccination status, symptoms and level of Ct-value.

As portrayed in Table 2, univariate logistic regression analysis displayed that the variables of age group ≥40 years old, being Malay, having comorbidity, being a doctor, working in a hospital setting, being asymptomatic and having a Ct-value ≥ 30 revealed significant results (*p*-value < 0.05) among group of healthcare-associated infections, and, hence, these variables were further analysed in the multivariate logistic regression analysis. 

In this study, we performed multivariate logistic regression to determine the factors associated with COVID-19 healthcare-associated infections. As illustrated in Table 2, doctors had 1.433 higher odds of getting COVID-19 healthcare-associated infection. as compared to administrative staff (AoR = 1.433; 95% CI = 1.044, 1.968). The model also revealed that working in a hospital setting gave rise to 1.439 more likelihood of acquiring COVID-19 healthcare-associated infection, as compared to working in a non-hospital setting (AoR = 1.439; 95% CI = 1.080, 1.917). In addition, the odds of acquiring asymptomatic COVID-19 healthcare-associated infection were 1.848, as compared to symptomatic (AoR = 1.848; 95% CI = 1.604, 2.130). Incomplete or non-vaccination was associated with being 1.400 more likely to acquire COVID-19 healthcare-associated infection, as compared to the vaccinated group (AoR = 1.400; 95% CI = 1.050, 1.866). Finally, a CT-value ≥ 30 indicated 2.494 higher odds of COVID-19 healthcare-associated infection, compared to a CT-value < 30 (AoR = 2.494; 95% CI = 1.927, 3.226).

The Hosmer and Lemeshow goodness of fit test was applied to the final model and the result of *p* = 0.1683 indicated that the model was a good fit. The model also correctly classified 73% of the respondents. According to Hosmer et al. (2013), an area under the ROC curve of more than 0.7 is considered acceptable discrimination. The area under the ROC curve for the model in this study was 0.7107, which meant that the model was able to discriminate 71.07%. We can, thus, conclude that the final model was achieved [16].

## 4. Discussion

The study aimed to determine the factors associated with healthcare-associated COVID-19 infection among healthcare workers in Kedah. In this study, we identified 554 cases of healthcare-associated infection. corresponding to 33.0% of all COVID-19 cases among healthcare workers in Kedah. The percentage was slightly lower than a study by Thibon et al. in France. which recorded 45% healthcare-associated cases [17]. In the United States, the Morbidity and Mortality Weekly Report (MMWR, April 2020) reported 55% of HCWs acquired COVID-19 due only to healthcare exposure [18]. In contrast, Singapore and the Netherlands reported lower percentages of HCWs infected with COVID-19 due to workplace exposure, at 16.7% and 22%, respectively [9,19]. The findings differed due to the number of total cases recorded during the study period in that particular country. It is estimated that there were higher weekly cases in the US (60k) and France (30k), compared to Malaysia (800), the Netherlands (600) and Singapore (41) [20,21,22,23,24]. Additionally, England (March 2020), recorded 240 HCWs infected with COVID-19, 1716 were recorded in Wuhan (until 11 February, 2020), and 843 in Italy (from March to April, 2020) [25,26,27]. However, it was not specified whether the cases were linked to healthcare-related exposure.

In the multivariate logistic regression, our study found that factors associated with healthcare transmission were being asymptomatic, not being vaccinated or having incomplete vaccination, a Ct-value ≥ 30 and being a doctor working in a hospital setting. A meta-analysis by Ma et al. reported the pooled percentage of asymptomatic infections among confirmed COVID-19 cases in the community was 40.5%. The percentage was even higher, at 47.53%, among nursing home residents and staff [28]. The high percentage of asymptomatic infections emphasise the potential transmission risk of asymptomatic infections. Our study confirmed that asymptomatic HCWs were a risk factor for transmission of COVID-19 in healthcare settings. Therefore, based on the study’s findings, asymptomatic HCWs should be tested and isolated if confirmed positive to reduce the risk of COVID-19 transmission. In the case of shortage of staff, risk assessment should be done, whereby HCWs with asymptomatic medium risk exposure should be allowed to return to work only if their first results were negative [13].

Second, our results showed that the risk factor for healthcare-associated infection was a Ct-value of more than 30. This indicated a lower quantity of viral load present in the sample, that the exposure was old and the virus isolation was later, as compared to the symptom onset. Therefore, risk assessment and contact tracing should be conducted as early as possible during the high Ct-value phase for early screening and isolation to prevent further transmission of COVID-19 in healthcare facilities.

Third, our results showed that not being vaccinated, or incompletely vaccinated, was one of the risk factors contributing to healthcare-associated infection. It was well established that the COVID-19 vaccination played a critical role in reducing COVID-19 transmission and disease severity. Hence, it is very important to ensure high vaccination coverage among healthcare workers who are frontliners in combating COVID-19 infection.

Fourth, our findings highlighted that working as a doctor in a hospital setting was a risk factor for healthcare-associated COVID-19 infection. This was in contrast to other studies that reported nurses, support service staff and administrative staff were at higher risk of contracting COVID-19 [29,30]. The findings could be due to the fact that doctors in hospital settings tended to have longer working hours and were exposed to critically ill patients requiring aerosol generating procedures, and. therefore, these doctors were at higher risk of contracting healthcare-associated COVID-19 [31]. Furthermore, the enclosed and crowded working environment in the hospital setting, such as air-conditioned wards, on-call rooms and pantries, could be contributory factors, as compared to working in health clinics, which tend to be more open and naturally ventilated. This finding was compatible with a study in Switzerland, conducted by Canova et al., that reported low risk of COVID-19 healthcare-associated transmission in primary care settings [32]. Therefore, continuous training and infection prevention control measures should be strengthened in hospital settings, such as practicing new norms, social distancing, and wearing appropriate PPE, as these remain the key strategies in preventing healthcare-associated transmission.

This study had limitations. Firstly, there was a possibility that the source of the infection of healthcare-associated infection was community acquired and vice versa. This classification bias was minimized by ensuring thorough investigation to identify the source of infection. Next, some important variables were not included in this study, such as training received, compliance to appropriate PPE and the practicing of infection prevention control measures.

## 5. Conclusions

The study reported that 33% of the COVID-19 cases among HCWs in Kedah were classified as healthcare-associated. We found the factors associated with healthcare-associated infection to be being asymptomatic, having incomplete vaccination, having a CT-value ≥ 30, being a doctor and working in a hospital setting. The identified factors would help in planning control measures to prevent healthcare-associated transmission. A good surveillance system regarding testing and reporting, and isolating and vaccinating remain key strategies in protecting HCWs and ensuring continuity of delivery of health systems. However, non-healthcare-associated infection was responsible for more than half of the COVID-19 infection cases among the HCWs, and, thus, further study should be done to identify high-risk behaviour. It is crucial to improve awareness among HCWs on COVID-19 exposure and its associated factors that contribute to high risk of infection. The impact of infected HCWs on the health system could be seen in absenteeism and quarantined staff, leading to a shortage of staff in health settings, which, thus, affected the health care delivery systems.

## Figures and Tables

**Table 1:** Socio-demographic characteristics of study participants (N = 1679).

Variables	Total N (%)	Non-Healthcare-Associated (n = 1125)	Healthcare-Associated (n = 554)	*p*-Value
Age group				**0.007**
<40 years	1154 (68.7)	749 (66.6)	405 (73.1)	
≥40 years	525 (31.3)	376 (33.4)	149 (26.9)	
Gender				0.099
Male	537 (32.0)	345 (30.7)	192 (34.7)	
Female	1142 (68.0)	780 (69.3)	362 (65.3)	
Ethnicity				**0.018**
Malay	1495 (89.0)	1016 (90.3)	479 (86.5)	
Non-Malay	184 (11.0)	109 (9.7)	75 (13.5)	
Comorbid				**0.005**
Yes	264 (15.7)	210 (18.7)	73 (13.2)	
No	1415 (84.3)	915 (81.3)	481 (86.8)	
Job Category				**0.001**
Doctor	291 (17.3)	166 (14.7)	125 (22.5)	
Nurses	618 (36.8)	424 (37.7)	194 (35.0)	
Assistant Medical Officer	113 (6.7)	76 (6.8)	37 (6.7)	
Pharmacist	39 (2.3)	24 (2.1)	15 (2.7)	
Dental officer	55 (3.3)	43 (3.8)	12 (2.2)	
Allied Health	75 (4.5)	60 (5.3)	15 (2.7)	
Ancillary staff	406 (24.2)	275 (24.4)	131 (23.6)	
Administrative staff	82 (4.9)	57 (5.1)	25 (4.5)	
Workplace setting				**0.009**
Hospital setting	1258 (74.9)	821 (73.0)	437 (78.9)	
Non-hospital setting	421 (25.1)	304 (27.0)	117(21.1)	
Reason of screening				**<0.001 ^a^**
Contact with family member or friend	469 (27.9)	464 (41.2)	5(0.9)	
Contact with patient/work colleague	489 (29.1)	4 (0.4)	485 (87.6)	
Symptomatic screening	616 (36.7)	611 (54.3)	5 (0.9)	
Targeted screening *	105 (6.3)	46 (4.1)	59 (10.7)	
Managing COVID-19				0.594
Yes	134 (8.0)	87 (7.7)	47 (8.5)	
No	1545 (92.0)	1038 (92.3)	507 (91.5)	
Completed vaccine				**<0.001**
Yes	1311 (78.1)	913 (81.2)	398 (71.8)	
No	368 (21.9)	212 (18.8)	156 (28.2)	
Symptomatic				**<0.001**
Yes	1261 (75.1)	947 (84.2)	314 (56.7)	
No	418 (24.9)	178 (15.8)	240 (43.3)	
Ct-value (n = 1515) **				**<0.001**
<30	1095 (72.3)	837 (80.4)	258 (54.4)	
≥30	420 (27.7)	204 (19.6)	216 (45.6)	

* e.g., mass screening due to health facilities cluster, pre-placement of new staff, pre-hospital admission, living in Enhanced Movement Control Order (EMCO) area; ** totals for the Ct value variable did not sum up to 1679, due to missing data; ^a^ Fisher exact was performed due to some cells containing less than 5, bold font indicates statistical significance (*p*-value < 0.05).

**Table 2:** Univariate and multivariate logistic regression on factors associated with healthcare-associated COVID-19 infection among HCWs in Kedah.

Variables	Crude OR (95% CI)	*p*-Value	Adjusted OR (95% CI)	*p*-Value
Age group				
<40 years				
≥40 years	0.732 (0.585, 0.917)	**0.007**	0.8537 (0.650, 1.120)	0.254
Gender				
Male			-	-
Female	0.833 (0.671, 1.035)	0.099	-	
Ethnicity				
Non-Malay				
Malay	0.685 (0.501, 0.937)	**0.018**	0.905 (0.621, 1.320)	0.607
Comorbid				
No				
Yes	0.661 (0.495, 0.882)	**0.005**	0.820 (0.592, 1.137)	0.236
Job Category				
Administrative staff				
Doctor	1.716 (1.016, 2.900)	**0.043**	1.433 (1.044, 1.968)	**0.026**
Nurses	1.043 (0.632, 1.719)	0.868	-	
Assistant Medical Officer	1.11 (0.601, 2.048)	0.739	-	
Pharmacist	1.525 (0.641, 3.165)	0.385	-	
Dental officer	0.636 (0.287, 1.407)	0.264	-	
Allied Health	0.570 (0.273, 1.189)	0.134	-	
Ancillary staff	1.086 (0.649, 1.816)	0.753	-	
Type of health facilities				
Non-Hospital setting				
Hospital setting	1.383 (1.0849, 1.763)	**0.009**	1.439 (1.080, 1.917)	**0.013**
Symptomatic				
Yes				
No	2.016 (1.795, 2.264)	**<0.001**	1.848 (1.604, 2.130)	**<0.001**
Vaccine				
Yes				
No/incomplete	1.688 (1.330, 2.141)	**<0.001**	1.400 (1.050, 1.866)	**0.022**
CT value				
<30		**<0.001**		
≥30	3.435 (2.710, 4.352)		2.494 (1.927, 3.226)	**<0.001**

Summary of model fit for final model: Hosmer Lemeshow goodness of fit value: *p*-value = 0.1683; Classification table: 73%; Area under the curve: 0.7107; 1.0 reference for all covariates of the table, bold font indicates statistical significance (*p*-value < 0.05).

## Data Availability

Data available on request from the authors.
